# Mu Rhythm Desynchronization while Observing Rubber Hand Movement in the Mirror: The Interaction of Body Representation with Visuo-Tactile Stimulation

**DOI:** 10.3390/brainsci13060969

**Published:** 2023-06-19

**Authors:** Satoshi Shibuya, Yukari Ohki

**Affiliations:** Department of Integrative Physiology, School of Medicine, Kyorin University, Tokyo 181-8611, Japan

**Keywords:** body ownership, rubber hand illusion (RHI), event-related desynchronization (ERD), mu-rhythm, electroencephalography (EEG), hand movement, embodiment

## Abstract

During rubber hand illusion (RHI), participants feel that a rubber (fake) hand is their own (i.e., embodiment of the rubber hand) if the unseen real hand and seen rubber hand are stroked synchronously (i.e., visuo-tactile stimuli). The RHI is also evoked if the real and rubber hands are placed in the same position (i.e., visual-proprioceptive congruency), which can be performed using a mirror setting. Using electroencephalography (EEG) and mirror settings, we compared μ rhythm (8–13 Hz) event-related desynchronization (ERD; an index of sensorimotor activation) while watching the movements of embodied or non-embodied rubber hands, which was preceded by an observation of the rubber hand with or without synchronous visuo-tactile stimuli. The illusory ownership of the fake hand was manipulated using visual continuity with (RHI) and without (non-RHI) a fake forearm. Resultantly, an ownership-dependent μ rhythm ERD was found when delivering visuo-tactile stimuli; a greater and more persistent μ rhythm ERD during the rubber hand movement was identified in the RHI in comparison to the non-RHI condition. However, no difference was observed between the two when observing the fake hand alone. These findings suggest the possibility that a self-related multisensory interaction between body representation (top-down processing) and visuo-tactile inputs (bottom-up processing) before a fake hand movement produces ownership-dependent sensorimotor activations during subsequent movement observations.

## 1. Introduction

A sense of body ownership is the experience that one’s body belongs to oneself and is a crucial aspect of self-awareness [1]. For healthy participants, body ownership has been intensively examined using bodily illusions such as the rubber hand illusion (RHI) [2]. In the classical visuo-tactile RHI paradigm, the participant perceives a life-sized fake (rubber) hand as their own when an unseen participant’s hand and the visible fake hand, which is placed near the real one, are stroked in synchrony (i.e., visuo-tactile stimulation). In contrast, an asynchronous visuo-tactile stimulation of the two hands or a synchronous visuo-tactile stimulation of an anatomically incongruent fake hand [3] and a non-body object (e.g., wooden block) [4] attenuates or abolishes the RHI, indicating that a multisensory integration of vision, touch, and proprioception (i.e., bottom-up factor) and a consistency between sensory inputs and the preexisting hand representation (i.e., top-down factor) are important for evoking this illusion [5]. To assess the RHI magnitude, many studies have used questionnaires, proprioceptive drift (i.e., a shift in the proprioceptive judgment of one’s own hand toward the fake one), and physiological measurements such as skin conductance response (SCR) to threaten the embodied fake hand. The relationship between ownership and embodiment has been considered in different ways [6]: by conceiving the two to be synonymous or by considering ownership as a subcomponent of embodiment (e.g., self-location and agency) [7]. Although we are sympathetic to the latter notion, this paper will use the term(s) “embodied fake hand” or “embodiment of fake hand” synonymously with “self-attributed fake hand” or “self-attribution of fake hand” (i.e., ownership) hereafter.

Illustrations of fake hands have also been explored in immersive virtual reality (VR) environments [8,9,10]. In the classical RHI setup in physical reality, it is impossible to completely match the spatial positions of the fake and real hands, resulting in discrepancies between proprioceptive (i.e., real hand) and visual (i.e., rubber hand) information. In contrast, one of the advantages of using VR stimulations is that the virtual and real hands can be placed in the same position, where the visuo-proprioceptive information is congruent. Under such circumstances, a mere observation of the virtual hand without visuo-tactile stimuli (i.e., brush strokes) can induce an illusory ownership of the observed virtual hand (i.e., virtual hand illusion, VHI) [11,12,13]. Several VR studies have reported that visual discontinuity between the virtual hand and body (e.g., missing a wrist) attenuates the VHI [12,13,14], regardless of the visual-proprioceptive congruency of the virtual and real hands. These findings suggest that the visual discontinuity paradigm is a useful method for assessing the effects of preexisting body representations on the sense of body ownership, without any spatial and postural mismatches between body and space [13].

Using the visuo-tactile RHI, recent behavioral and electrophysiological studies have investigated the effects of observing embodied fake hand movements on observers’ illusory kinesthesia (i.e., sensing movement) [15] and sensorimotor brain activities [16,17,18,19]. For instance, it was found that watching lateral or sagittal movements of the embodied fake hand induced illusory movements of the real hand in the same movement (i.e., kinesthetic illusion) [15]. Using electroencephalography (EEG) and RHI, our group compared the effects of observing embodied fake hand movements on the observer’s sensorimotor system [16,17,18]. We focused on mu (μ) rhythm event-related desynchronization (ERD) to evaluate these sensorimotor activations, because the oscillation power of the μ frequency band (8–13 Hz), which is predominantly recorded above the central electrodes (C3, C4, or Cz), is decreased during motor execution and movement observation [20,21]. These results suggest a greater μ rhythm ERD during movement observations of the embodied hand compared to the non-embodied fake hand. This phenomenon was replicated in an immersive VR study [19], in which a greater μ rhythm ERD was detected when viewing the movements of the embodied virtual hand, as compared to the non-embodied hand or non-body object.

Related studies have demonstrated that watching a video of someone else’s moving hand evokes illusory kinesthetic sensations and corticomotor excitability when the video image is set over a real hand [22]. A functional magnetic resonance imaging (fMRI) study also reported a larger activation in components of the human mirror neuron system, including the left dorsal and ventral premotor cortices and left superior and inferior parietal lobules, when participants observed a video image of their own hand movement compared to that of another person’s hand movement [23]. A following EEG study showed that kinesthetic illusions via video movies of one’s own moving hand were associated with a greater μ rhythm ERD [24]. While these studies did not use the RHI method to manipulate hand ownership, the findings are interesting, as the mere observation of human hand movement can evoke the sensorimotor brain activations involved in kinesthetic illusions.

Previous studies investigating μ rhythm ERD whilst observing rubber hand movement have used the visuo-tactile RHI paradigm, wherein the spatial positions of the rubber hand and participant’s hand were different. Therefore, it remains unclear whether ownership-dependent μ rhythm ERD modulation would occur in a situation where the two hands are placed in the same position (i.e., visuo-proprioceptive congruence). Although we predicted that the mere observation of rubber hand movement would also elicit a μ rhythm ERD in this situation, we aimed to investigate the effects of the preexisting body representation (top-down processing) on the μ rhythm ERD. Additionally, another question we hoped to address was: how does synchronous visuo-tactile stimulation (bottom-up processing) under visuo-proprioceptive congruency influence μ rhythm ERD during rubber hand movement? Using an EEG, we compared the μ rhythm ERD evoked by the fake hand movement between after a mere observation of the embodied (or non-embodied) fake hand and after the delivery of synchronous visuo-tactile stimulation. To achieve a visual-proprioceptive congruency of the fake and real hands in order to induce the RHI, we used a mirror setting rather than immersive VR. In this experiment, the mirror was positioned along the midsagittal plane and the fake and real hands were aligned equidistantly from the mirror on either side, resulting in a reflection of the fake hand being projected onto the position of the participant’s unseen real hand [25]. According to previous findings [11,12,13,14], the ownership of the fake hand in the mirror was manipulated by the visual discontinuity between the fake hand and the participant’s body (i.e., missing a forearm). In addition to being an assessment of body representation, the visual discontinuity paradigm is useful because it makes visual information from the fake hand remain constant during movement observation. As a result, the μ rhythm ERD was modulated by illusory hand ownership when delivering visuo-tactile stimuli, but not when observing the fake hand alone. Our prediction based on these results is that a multisensory interaction between body representation and visuo-tactile inputs before fake hand movement would elicit an ownership-dependent μ rhythm ERD during subsequent movement observations.

## 2. Materials and Methods

### 2.1. Participants

Eighteen healthy individuals (12 men and 6 women; age: mean ± standard deviation, 23.1 ± 4.0 years) participated in this study. The participants were blinded to the purpose of the experiment. Sixteen participants were strongly right-handed (L.Q. (laterality quotient) > +90) and the remaining two were left-handed (L.Q. = –50 and –80) according to the Edinburgh Handedness Inventory [26].

### 2.2. Apparatus

**Figure 1:**
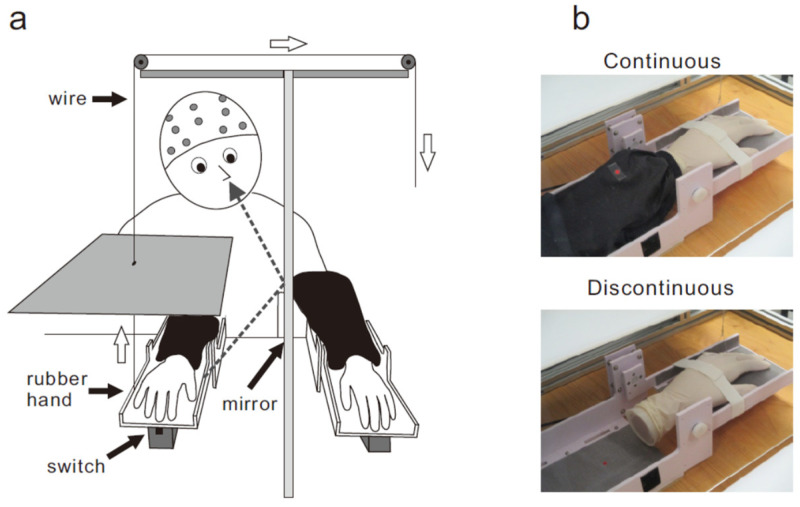
(**a**) Experimental setup. (**b**) A fake limb in the mirror (top: continuous (Con) condition; bottom: discontinuous (Dis) condition).

The participants were seated in front of a wooden desk and fitted with a white latex glove and black arm cover on their left hand and forearm, respectively, which were placed on custom-made equipment (WMP-1002, Uchida Electronics Co., Tokyo, Japan) to measure their wrist angles (Figure 1a). A life-sized fake (right) hand and forearm with identical latex gloves and arm covers were placed on the same equipment in front of the participants (Figure 1b, top; congruent conditions; see Procedure). Under discontinuous conditions (see below), the fake forearm was removed (Figure 1b, bottom). Both the fake and real hands were fixed to the manipulandum using Velcro tape. To prevent the participants from seeing the fake upper limb, a white acrylic board (30 × 45 cm) was positioned 20 cm above the fake hand. The distance between the real and rubber hands was maintained at 36 cm throughout the experiment. A half-mirror (43 × 50 cm) was placed vertically between the real and fake middle fingers, such that the spatial location of the reflected visual image (i.e., the fake right arm) in the mirror was completely identical to that of the real arm. Because the front part of the equipment was attached to a thin wire, the experimenter could lift the fake hand (i.e., the wrist extension of the fake arm) by pulling the wire downward (see white arrows in Figure 1a). A mechanical switch (STF15; Asa Electronics Industry Co., Tokyo, Japan), attached immediately beneath the equipment, was used to detect the initiation of fake hand movement. We used the time of the signal input from the switch as a trigger event in the EEG analysis. 

### 2.3. Procedure

**Figure 2:**
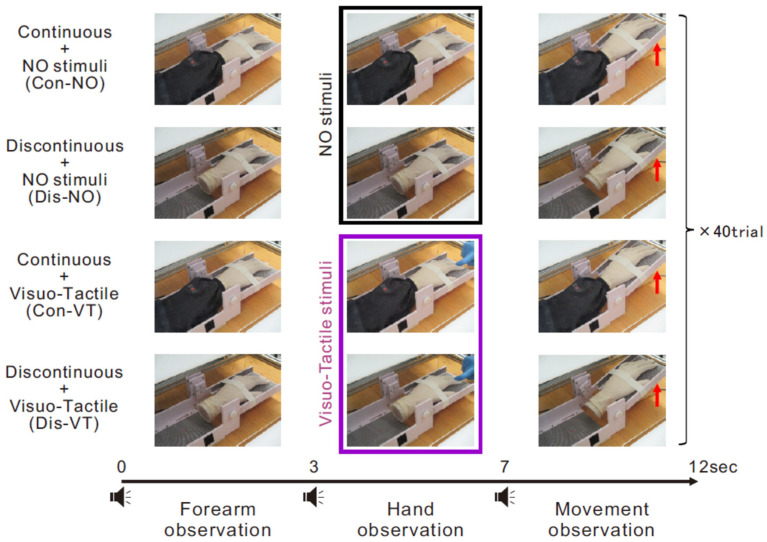
Experimental flow of one trial for all conditions (i.e., Con-NO, Dis-NO, Con-VT, and Dis-VT conditions). Each trial included three phases: *Forearm observation* (3 s), *Hand observation* (4 s), and *Movement observation* (5 s) phase. While visuo-tactile stimuli were delivered during hand observation phase in the VT conditions (Con-VT and Dis-VT: purple rectangle), no stimuli were delivered in the NO conditions (Con-NO and Dis-NO: black rectangle).

Each participant experienced four experimental conditions with a 2 × 2 factorial design: body continuity (continuous (Con) vs. discontinuous (Dis)) and stimuli (no stimuli (NO) vs. visuo-tactile stimuli (VT)). According to this combination, we refer to the four conditions as the Con-NO, Dis-NO, Con-VT, and Dis-VT conditions hereafter. The order of the four conditions was randomized across the participants. Each condition comprised 40 trials and each trial included three phases, according to a previous VR-based study involving visual discontinuity [13]: forearm observation, hand observation, and movement observation (Figure 2). One of the purposes of the forearm observation phase was to make the participants aware of the absence (or presence) of their forearm. The shift timing between the phases was informed by the delivery of auditory cues (0.5 kHz (forearm observation), 1.0 kHz (hand observation), and 0.75 kHz (movement observation) pure tones). During the forearm observation phase (3 s), the participants were required to fixate on a small red dot (0.8 cm in diameter) placed on a specific location of the fake forearm (continuous conditions (Con-NO and Con-VT)) or the corresponding location of the equipment (discontinuous conditions (Dis-NO and Dis-VT)). During the hand observation phase (4 s), the participants shifted their gaze from the fake forearm to the fake hand and an experimenter stroked the fake hand’s and participant’s fingers twice (i.e., the experimenter stroked two index fingers at first (first stroking) and then stroked two middle fingers (second stroking)) in synchrony, using their right and left index fingers at approximately 1.0 Hz in the visuo-tactile stimuli conditions (Con-VT and Dis-VT). In contrast, under the no-stimulus condition (Con-NO and Dis-NO), the participants continued to watch the fake hand. In the movement observation phase (5 s), the participants observed that the wrist of the fake hand in the mirror extended to approximately 15° for 1 s at an almost constant speed (the fingertips rose by approximately 6 cm), and then returned to the starting position for 1 s, with the experimenter pulling the wire.

Following the completion of each condition, the participants reported their subjective feelings during the task using a questionnaire. The questionnaire comprised nine items (Table 1). Eight items (Q1 to Q8) were selected based on previous studies [2,13,27], whereas Q9 was used to assess whether the participants felt their own hand to be moving while observing the fake hand movements (i.e., illusory kinesthesis). The participants responded to all the items using a 7-point Likert scale ranging from +3 (strongly agree) to −3 (strongly disagree). The nine items were classified into five categories: *ownership* (Q1 and Q2), *ownership control* (Q3 and Q4), *agency* (Q5 and Q6), *agency control* (Q7 and Q8), and *motor awareness* (Q9). Except for *motor awareness*, the ratings obtained were averaged for each category. The participants were allowed 5 min intervals between the conditions.

### 2.4. Acquisition and Analysis of EEG Data

During the experiment, continuous EEG signals were recorded from 16 international and 10–20 system locations (Fp1, Fp2, F3, F4, F7, F8, Cz, C3, C4, T7, T8, P3, P4, P7, P8, and O2) at 125 Hz using a mobile EEG signal amplifier (Cyton + Daisy; OpenBCI, New York, NY, USA). The EEG impedance was maintained below 15 kΩ during the experiment. The reference electrode was located on the participant’s left earlobe and the ground electrode was positioned at AFz. The trigger onset was defined as the time of the movement initiation of the fake hand in the mirror. EEGLAB (version 2022.0) was used for the data analysis [28]. For the data preprocessing, raw data were applied to a 1–40 Hz band-pass filter. Subsequently, the data were segmented into epochs from 0.5 s pre-onset to 2.5 s post-onset. Bad epochs were discarded according to predetermined criteria, which was as follows: (1) an epoch should include an amplitude exceeding ± 100 μV; (2) the epoch should include a trend exceeding 50 μV/epoch; and (3) the epoch should include a power spectrum exceeding five standard deviations from the mean. In this epoch rejection, we did not remove the epochs that included eye movement, eye blinks, and electromyogram (EMG), because these components could be isolated in the next step (i.e., independent component analysis (ICA)). Accordingly, 1.3 ± 1.5 (mean ± SD), 1.2 ± 1.4, 1.2 ± 1.8, and 1.6 ± 2.2 epochs out of 40 were discarded in the Con-NO, Con-VT, Dis-NO, and Dis-VT conditions, respectively.

After the epoch rejection, an independent component analysis (ICA) was applied to the preprocessed EEG data using the EEGLAB “runica” function (InfoMax ICA algorithm) [29]. We excluded components reflecting eye movements and other artifacts using the MARA (multiple artifact rejection algorithm) plugin for EEGLAB (version 1.2) [30].

For the time-frequency analysis, we used event-related spectral perturbation (ERSP), which measures the average dynamic changes in the amplitude of the broadband EEG frequency spectrum as a function of time relative to an experimental event [31]. The ERSP during the *movement observation* phase, in comparison to the baseline (−0.5 to 0 s from onset), was computed using Morlet wavelet transform. Then, the ERSP values were averaged in the μ frequency range (8–13 Hz) to investigate the μ-rhythm ERD. Permutation tests were performed to assess the significant differences in the ERSP data between the conditions at *p* < 0.05 and corrected for multiple comparisons using false discovery rate control.

## 3. Results

### 3.1. Questionnaire Rating

**Figure 3:**
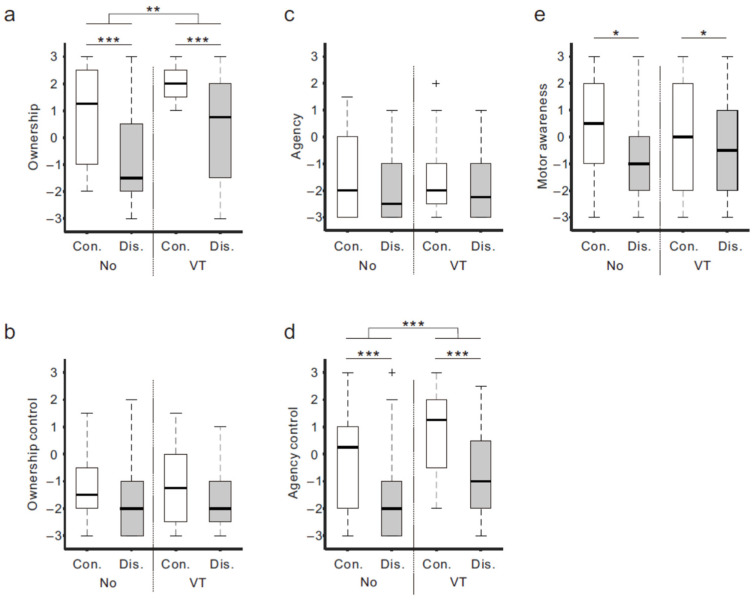
Questionnaire ratings. Boxplots of questionnaire ratings for the four experimental conditions are presented for *ownership* (**a**), *ownership control* (**b**), *agency* (**c**), *agency control* (**d**), and *motor awareness* (**e**). Boxes (white: continuous (Con) condition; gray: discontinuous (Dis) condition) and thick lines denote the interquartile ranges (IQRs) and medians, respectively. Whiskers represent either additional data points or extend to 1.5 × IQR. Small plus signs indicate outliers (values beyond 1.5 × IQR). Significance is denoted using asterisks (* *p* < 0.05, ** *p* < 0.01, and *** *p* < 0.001).

The boxplots in Figure 3 show the medians and interquartile ranges of the questionnaire ratings of the four conditions for the five categories (a–e). For *ownership* (Figure 3a), a two-way analysis of variance (ANOVA) demonstrated significant main effects of body continuity (Con vs. Dis; F _(1,17)_ = 27.3, *p* < 0.001) and stimuli (No vs. VT; F _(1,17)_ = 10.5, *p* < 0.01), but no interaction (*p* > 0.9). The rating of the Con condition (white box) was higher than that of the Dis condition (gray box) under both the NO (Con-NO (median score, 1.25) vs. Dis-NO (−1.5)) and VT (Con-VT (2.0) vs. Dis-VT (0.75)) conditions. In addition, the ratings of the VT conditions were greater compared to the NO conditions. For *ownership control* (Figure 3b) and *agency* (Figure 3c), overall, the ratings were considerably low (medians < −1) and there was no statistically significant difference between the conditions. In *agency control* (Figure 3d), the ANOVA showed that both main effects were significant (body continuity: F _(1,17)_ = 24.4, *p* < 0.001; stimuli: F _(1,17)_ = 16.6, *p* < 0.001). Like the *ownership* rating, a greater rating of the Con condition compared to the Dis condition was identified under both the stimulus conditions (NO: Con-NO (0.25) vs. Dis-NO (−2.0)) (VT: Con-VT (1.25) vs. Dis-VT (−1.0)). Moreover, the ratings of the VT conditions were greater compared to the NO conditions. Regarding *motor awareness* (Figure 3e), only the main effect of body continuity was significant (F _(1,17)_ = 5.99, *p* < 0.05), suggesting that the ratings of the Con conditions (Con-NO (0.25) and Con-VT (0)) were higher than the Dis conditions (Dis-NO (−1.0) and Dis-VT (−0.5)).

### 3.2. EEG Results

**Figure 4:**
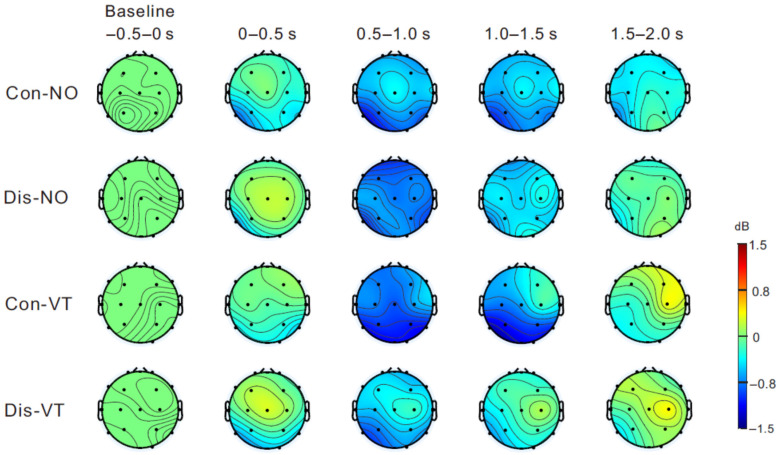
Mean topographies of event-related desynchronization (ERD) from the fake hand movement onset to 2 s post-onset in steps of 0.5 s. Topographic maps are shown in the frequency of the μ rhythm range (8–13 Hz). Blue color indicates decreased power.

**Figure 5:**
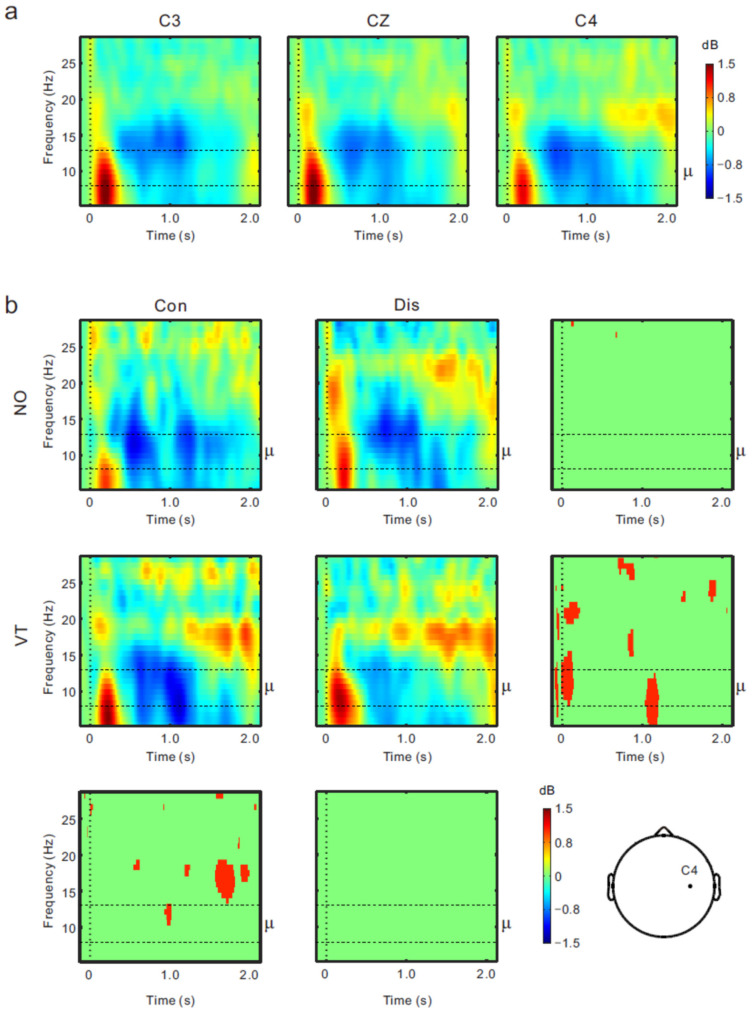
(**a**) Event-related spectrum perturbation (ERSP) time-frequency plots across conditions at the C3, Cz, and C4 positions. (**b**) ERSP time-frequency plots for the Con-NO (upper left), Dis-NO (upper middle), Con-VT (middle left), and Dis-VT (middle) conditions at the C4 position. Four panels around ERSP plots show statistically significant difference in time-frequency plots at the *p* < 0.05 level (permutation tests) between the Con-NO and Dis-NO conditions (upper right), the Con-VT and Dis-VT conditions (middle right), the Con-NO and Con-VT conditions (lower left), and the Dis-NO and Dis-VT conditions (lower middle).

Figure 4 shows the mean topographies of the μ rhythm power (8–13 Hz) from the fake hand movement onset to 2 s post-onset in steps of 0.5 s. An obvious μ rhythm ERD was widely observed over the sensorimotor regions from 0.5 to 1.5 s after the movement onset across all the conditions. Figure 5a shows the ERSP data across all the conditions at the C3 (left), Cz (middle), and C4 (right) positions. While a similar tendency was observed at all the central electrodes, the greatest and most persistent μ rhythm ERD (dark blue) was confirmed to be at the C4 electrode. Therefore, we compared the ERSP data between the conditions at the C4 electrode (Figure 5b). Permutation tests revealed the significant μ rhythm ERD difference between the Con-VT and Dis-VT conditions from 1.0 to 1.2 s post-onset (red area; *p* < 0.05).

**Figure 6:**
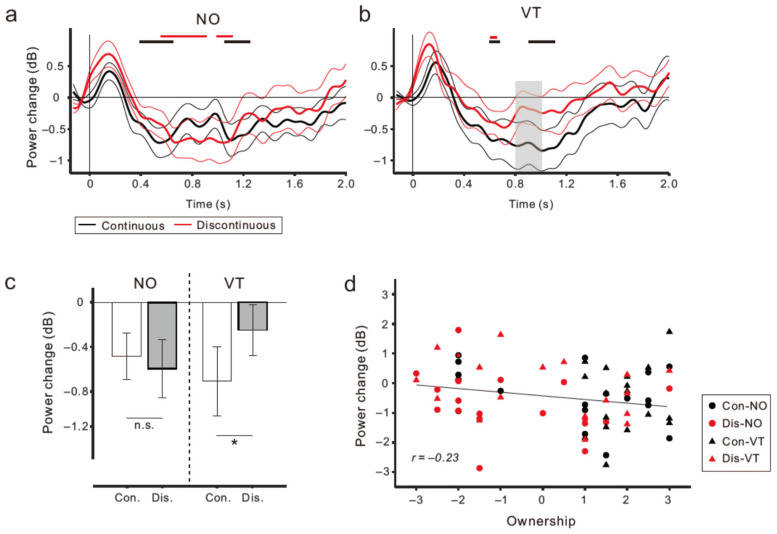
(**a**,**b**) Time courses of power reduction in the μ rhythm frequency range in the NO (left panel; (**a**) and VT (right panel; (**b**)) conditions, which are computed by the averaged data across the C3, C4, and Cz electrodes. Black and red lines denote the Con and Dis conditions, respectively. One thick and two thin lines denote the mean and ± 1.0 standard error (SE). A gray area in (**b**) represents an interval in which significant differences were identified between the Con and Dis conditions (*p* < 0.05; paired one-tailed *t*-test). (**c**) Mean power within μ rhythm frequency range from 0.6 to 1.3 s post-movement onset in each condition. Vertical lines denote ± 1.0 SE. Significance is denoted using an asterisk (* *p* < 0.05). n.s. means not significant. (**d**) Correlation analysis showing a marginally significant negative correlation between *ownership* rating and the magnitude of μ rhythm ERD (*r* = −0.23, *p* = 0.05).

Figure 6a,b depict the μ rhythm power changes averaged across the C3, C4, and Cz electrodes, based on the pre-movement baseline (−0.5 to 0 s) for the NO (left panel) and VT (right panel) conditions. One thick line and two thin lines represent the mean and its standard error (SE), respectively. It is worth noting that the transient power upticks seen immediately after the movement onset are artifacts due to the external trigger inputs (see also the ERSPs in Figure 5). After the transient power upticks (artifacts), the power gradually decreased from 0.4 s to 1.0 s post-onset and then returned to the baseline again. In the NO condition (Figure 6a), the time course of the power reduction was similar between the Con (Con-NO; black lines) and Dis (Dis-NO; red lines) conditions. In contrast, in the VT condition (Figure 6b), the power reduction in the Dis condition (Dis-VT, red) was much weaker and the start to return was earlier (0.7 s) than that in the Con condition (1.1 s) (Con-VT, black). A gray area in Figure 6b shows the intervals in which significant differences were identified between the two conditions (*p* < 0.0.05; paired *t*-test). The upper horizontal lines in Figure 6a,b denote the intervals in which the power was significantly decreased relative to the baseline. Considering such intervals, we further computed the mean μ rhythm power (averaged across C3, C4, and Cz) from 0.6 s to 1.3 s post-onset for each condition (Figure 6c). As a result of the two-way ANOVA, only the interaction between body continuity and stimuli was significant (F _(1,17)_ = 4.8, *p* < 0.05). A subsequent analysis showed that the difference in the power reduction magnitude was significant under the VT conditions (Con-VT vs. Dis-VT: *p* < 0.03, Fisher’s least significant difference test), but not under the NO conditions (Con-NO vs. Dis-NO: *p* > 0.5).

We performed a correlation analysis to assess the relationship between the μ rhythm ERD magnitude (averaged across C3, C4, and Cz) from 0.6 s to 1.3 s post-onset and the strength of the illusory hand ownership (*ownership* rating). First, the analysis was conducted using data (36 pairs) from the Con (Con-NO and Con-VT; black circles and triangles in Figure 6d) and Dis (Dis-NO and Dis-VT; red circles and triangles) conditions, respectively (i.e., RHI vs. non-RHI). The results showed no significant relationship in either condition (Con: *r* = −0.20, *p* > 0.24; Dis: *r* = −0.23, *p* > 0.1; Pearson’s product moment correlation coefficient). When the pooled data across all the conditions (72 pairs) were used, a weak and marginally significant negative correlation was identified (*r* = –0.23, *p* = 0.05). 

**Figure 7:**
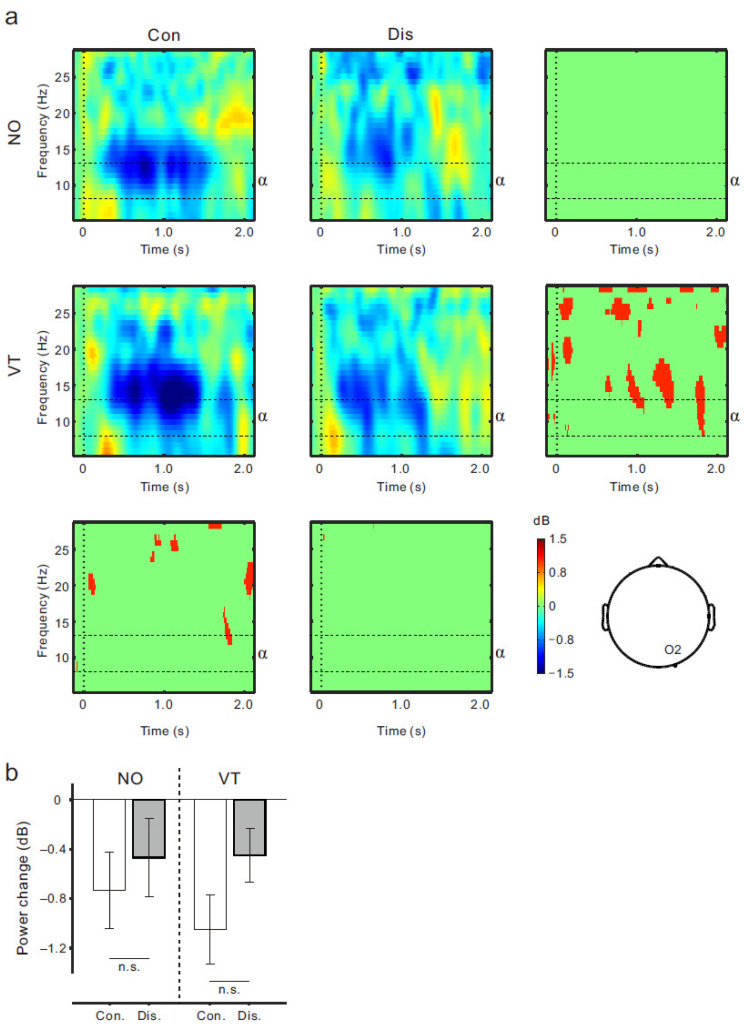
(**a**) ERSP time-frequency plots for the Con-NO (upper left), Dis-NO (upper middle), Con-VT (middle left), and Dis-VT (middle) conditions at the O2 electrode. Four panels around ERSP plots show statistically significant difference in time-frequency plots at the *p* < 0.05 level (permutation tests). (**b**) Mean power within α rhythm frequency range (8−13 Hz) from 0.6 s to 1.3 s post-movement onset in each condition. Vertical lines denote ± 1.0 SE. n.s. means not significant.

We further compared the ERSP data between the conditions at the O2 position (occipital electrode; Figure 7a) to check the effects of the posterior α rhythm on the μ rhythm ERD results. When using the same intervals (0.6–1.3 s post-onset) and frequency band (8–13 Hz), no obvious difference of the α rhythm ERD was found between the conditions, although there seemed to be a difference (red areas, Figure 7a). The two-way ANOVA demonstrated that the main effects of the body continuity (_)_ = 3.1, *p* > 0.09), stimuli (F _(1,17)_ = 0.37, *p* > 0.5), and their interaction (F _(1,17)_ = 0.46, *p* > 0.5) were not significant (Figure 7b).

## 4. Discussion

Using EEGs and a novel experimental setting with a mirror, we compared the μ rhythm ERDs during watching the movement of the illusory embodied (RHI; continuous (Con) condition) and non-embodied (non-RHI; discontinuous (Dis) condition) fake hand between after merely observing the fake hand (NO condition) and after delivering synchronous visuo-tactile stimulation (VT condition). A stronger illusory ownership over the fake hand was induced by visual continuity between the fake hand and the body (Con conditions), but was attenuated by visual discontinuity (Dis conditions). Overall, we found an obvious μ rhythm ERD from 0.6 s to 1.3 s after the movement onset. An ownership-dependent modulation of the μ rhythm ERD (Con vs. Dis) was found when delivering the visuo-tactile stimuli—a weaker and shorter μ rhythm ERD in the Dis-VT condition as compared to the Con-VT condition. However, no μ rhythm ERD difference was shown when merely observing the fake hand. These results suggest the possibility that a bodily self-related multisensory interaction between body representation (top-down processing) and visuo-tactile inputs (bottom-up processing) before the fake hand movement produced ownership-dependent sensorimotor activities during subsequent movement observations.

### 4.1. Μu Rhythm ERD and Sense of Body Ownership

A difference in the μ rhythm ERDs between the continuous (RHI) and discontinuous (non-RHI) conditions was found only when delivering synchronous visuo-tactile stimuli to the seen fake hand and participant’s unseen hand (i.e., VT conditions): there were weaker sensorimotor responses in the Dis-VT condition as compared to the Con-VT condition. In fact, the *ownership* rating difference between the Con-VT and Dis-VT conditions was also significant. These results suggest that the ownership-dependent μ rhythm ERD during fake hand movement [16,17,18,19] can be replicated, even in situations where the real and fake hands are positioned similarly. However, we did not find a significant μ rhythm ERD difference between the Con-VT and Con-NO conditions, although the main effect of the stimuli was significant in the ownership rating. This result does not support the idea that synchronous visuo-tactile stimulation further enhances the sensorimotor brain activation evoked by embodied fake hand movements. Instead, it is natural to consider that the synchronous visuo-tactile stimulation of the non-embodied fake hand (i.e., the fake hand without the forearm; Dis-VT condition) suppressed these sensorimotor responses during the fake hand movement. In addition, the fact that we observed no effect of body discontinuity on the μ rhythm ERD in the NO conditions seems to support the notion that the ownership-dependent μ rhythm ERD in the VT conditions was indeed due to the multisensory interactions between the visuo-tactile (bottom-up) and body representation (top-down) processing before the movement observation. Our group proposes that this ownership-dependent μ rhythm ERD was driven by inter-sensory conflict between vision (“*my hand is moving*”) and proprioception (“*my hand is not moving*”). In such situations, the sensorimotor system must resolve this intersensory discrepancy by changing the participant’s hand position (i.e., proprioceptive inputs). While the present study did not observe the participant’s actual movements, previous studies have reported that a visuo-proprioceptive discrepancy between the illusory embodied fake hand and real hand can induce actual hand movements to compensate for the conflict [18,32,33], and this compensatory movement (or force) can be interpreted by the active inference framework [33]. Our assumption for the present results is that, when the visual and proprioceptive information of the fake and real hands was congruent (as in the current experiment), the synchronous visuo-tactile stroking of the non-embodied (detached) fake hand provided negative rather than positive feedback on visual–proprioceptive integration concerning the participant’s own body representation, resulting in sensorimotor activation (μ rhythm ERD) after the fake hand movement became smaller.

In contrast, we found no ownership-dependent μ rhythm ERD differences when merely observing the fake hand (i.e., Con-NO vs. Dis-NO), even though the questionnaire indicated a clear difference in the *ownership* ratings. This dissociation between body ownership and μ rhythm ERD may suggest a possibility that the visual appearance of a moving fake body part is more dominant than body ownership when sensorimotor activations are evoked by a mere observation of the fake body part, which is placed in the same position as the real one. In our experiment, to examine ownership-dependent μ rhythm ERDs using visual (dis)continuity between the fake hand and participant’s body, the visual appearance of the fake hand itself remained constant across all the conditions. Thereby, the visual information of the fake hand movement might have evoked similar sensorimotor responses. If a different visual appearance, such as a non-body object (e.g., wooden block) or anatomically incongruent fake hand, was used as a control (non-RHI) condition, an μ rhythm ERD difference might appear. The other possibility is that the μ rhythm ERD during the fake body movement occurred even if its visual appearance was not natural looking. This notion is partially consistent with the previous finding of an immersive VR study [13], which examined the relationship between illusory ownership over a virtual hand and the skin conductance response (SCR) associated with a threat approaching this virtual hand. The results suggest that inducing ownership over a virtual hand requires a natural-looking visual appearance of the virtual limb, whereas a non-natural looking visual appearance is accepted in the SCR response.

In summary, an ownership-dependent μ rhythm ERD was evoked when delivering visuo-tactile stimuli (Con-VT vs. Dis-VT), but not when merely observing the fake hand (Con-NO vs. Dis-NO). In the current study, hand ownership (RHI) was manipulated using visual (dis)continuity between a fake hand and the body, which is the modulation of top-down processing involved in the consistency between sensory inputs (i.e., vision and proprioception) and the preexisting body (hand) representation [5]. Therefore, the current results suggest that a self-related multisensory interaction between body ownership (top-down processing) and synchronous visuo-tactile inputs (bottom-up processing) before a fake hand movement would cause an ownership-dependent μ rhythm ERD (sensorimotor activation) during subsequent movement observations.

There are several reasons why the correlation between the *ownership* rating and μ rhythm ERD magnitude was relatively low. First, as described above, in the NO conditions, there was no μ rhythm ERD difference between the Con and Dis conditions, even though an obvious difference in the *ownership* rating was obtained. Second, the *ownership* ratings of the Dis conditions (i.e., Dis-NO and Dis-VT) showed a much higher inter-subject variability (Figure 3a). This result is consistent with the previous findings of a VR study [13]. Third, in some participants, greater μ rhythm event-related synchronization (ERS), rather than ERD, was observed (Figure 6d). Given that some prior studies have also failed to detect correlations between the μ rhythm ERD and a sense of hand ownership [17,19], the causal connection between the two appears tenuous, if there is any.

### 4.2. Sense of Agency and Motor Awareness during Movement Observation

A sense of agency refers to the subjective feeling of controlling one’s actions [1]. The questionnaire results showed that the *agency* ratings were quite low across all the conditions (medians < −2). This is not surprising, as the brain does not generate motor commands associated with voluntary movements [34,35]. However, a higher *agency control* rating was observed under the continuous (RHI) condition in comparison to the discontinuous condition (non-RHI) (Figure 3d). In particular, the *agency control* rating of the Con-VT condition was above +1 (median: +1.3), supporting the idea that the participants felt that the fake hand was controlling their hand movements during the movement observation. Moreover, the *agency control* and *ownership* ratings were highly correlated (*r* = 0.67, *p* < 0.001; *n* = 72). Considering these results, we assume that, when the participants strongly felt an illusory ownership of the fake hand (i.e., embodiment of the fake hand) but did not make voluntary movements, they experienced the illusion that their own hand was controlled by the fake hand while viewing the fake hand movement.

Because the statement of *motor awareness* (“*I felt as if my hand was moving against my will during observing the rubber hand movement*”) was similar to that used in previous studies investigating kinesthetic illusions [15], the relatively low *awareness* rating (median: −1 to 0.5) suggests that a stronger kinesthetic illusion did not occur in our study. This finding is seemingly inconsistent with previous studies, in which people experienced kinesthetic illusions when viewing videos of own-hand movement [24] and a motion of the embodied fake hand after the RHI [15]. However, the methodological differences between previous studies and our experiments should be considered. In previous studies, the rubber hand was moved continuously while the stroking (visuo-tactile stimuli) continued [15] and training (i.e., watching a video movie) to induce an adequate kinesthetic illusion was performed before the experiment [24]. Therefore, we inferred that observing continuous hand movements was necessary for evoking illusory kinesthesia. Another difference is that our *motor awareness* statement might include not only a sense of movement, but also a sense of agency due to the term “*against my will*”. However, considering that the motor awareness ratings differed from those of the agency and agency control, we assumed that the participants mostly responded to kinesthetic sensations during the movement observation.

### 4.3. Limitation 

The current study had some limitations. First, the EEG data were analyzed at the electrode level. A related study suggested that ERD at 10 Hz evoked by movement observation depends on attentional demand, because similar patterns of power reduction were found at the central and occipital electrodes [36]. Given the volume conduction, there is no denying the possibility that the μ rhythm ERD recorded from the sensorimotor region was contaminated by the posterior alpha rhythm ERD [37]. However, we analyzed the ERSP data at the O2 electrode (i.e., the occipital lobe) and found no difference between the conditions (Figure 7). Thus, we believe that the effects of this contamination on the μ rhythm ERD results were meagre. Second, unlike previous studies, the fake hand movement used in our experiment was conducted by moving the equipment rather than the fake hand itself. Such a fake hand movement with the equipment might have influenced the subjective experience during this movement. Third, the fake hand movement in our study was limited to wrist extension. It is possible that sensorimotor activation patterns during movement observation are dependent on movement type. Indeed, spreading the fingers of the fake hand induced μ rhythm ERD [17,18], whereas the fake hand rotation evoked not only μ ERD, but also beta ERD (15–25 Hz) [16]. Thus, it remains unclear whether the present findings could be replicated for other types of fake hand movements. Fourth, we used only two strokes of the fake and real fingers as the visuo-tactile stimuli. Although our EEG results demonstrated no μ rhythm ERD difference between the Con-VT and Con-NO conditions, it is possible that more frequent and long-term stroking (i.e., visuo-tactile stimulation) could enhance the sensorimotor activation while observing fake hand movements.

## 5. Conclusions

We compared the μ rhythm ERDs during watching embodied and non-embodied fake hand movements between after a mere observation of the fake hand and after delivering synchronous visuo-tactile stimulation. An ownership-dependent modulation of the μ rhythm ERD was found when the participants received visuo-tactile stimuli, but not when they only observed the fake hand. These findings may suggest that the multisensory interaction of body representation (top-down processing) with visuo-tactile inputs (bottom-up processing) causes ownership-dependent sensorimotor activation while observing fake hand movements.

## Figures and Tables

**Table 1:** Questionnaire comprising nine items that were classified into five categories.

Category	Question
Ownership	1. I felt as if I were looking at my own hand.
	2. I felt as if the rubber hand was my hand.
Ownership control	3. It seemed as if I might have more than one left hand or arm.
	4. It felt as if I no longer had a left hand, as if my left hand had disappeared.
Agency	5. I felt as if I was controlling the movements of the rubber hand.
	6. I felt as if I was causing the movements of the rubber hand.
Agency control	7. I felt as if the rubber hand was controlling my hand movements.
	8. I felt as if the rubber hand was controlling my will.
Motor awareness	9. I felt as if my hand was moving against my will when observing the rubber hand movement.

## Data Availability

The data presented in this study are available on request from the corresponding author.
